# Supplementary material for: Dynamic changes in the brain protein interaction network correlates with progression of Aβ42 pathology in Drosophila
Source: Sci Rep. 2020 Oct 28;10:18517. doi: 10.1038/s41598-020-74748-9 (PMC7595221; doi:10.1038/s41598-020-74748-9)
Supplement: Supplementary file 1 — Supplementary Information. [file 41598_2020_74748_MOESM1_ESM.docx]

Dynamic changes in the brain protein interaction network correlates with progression of Aβ42 pathology in *Drosophila*

Harry M. Scholes^1,*^, Adam Cryar^1,*^, Fiona Kerr^2,3,*^, David Sutherland^1^, Lee A. Gethings^4^, Johannes P. C. Vissers^4^, Jonathan G. Lees^1,5^, Christine A. Orengo^1,#^, Linda Partridge^2,6,#^, Konstantinos Thalassinos^1,#,$^

1 Institute of Structural and Molecular Biology, University College London, London, United Kingdom

2 Institute of Healthy Ageing, University College London, London, United Kingdom

3 Department of Biological and Biomedical Sciences, School of Health and Life Sciences, Glasgow Caledonian University, Glasgow, United Kingdom

4 Waters Corporation, Wilmslow, United Kingdom

5 Current address: Faculty of Health and Life Sciences, Oxford Brookes University, United Kingdom

6 Max Planck Institute for Biology of Ageing, Cologne, Germany

* Joint first authors

# Corresponding authors

$ Correspondence should be addressed to k.thalassinos@ucl.ac.uk

# Supplementary Information

## Methods

### IM-DIA-MS analysis

Nanoscale liquid chromatography (LC) separation of tryptic peptides was performed using a nanoAcquity UPLC system (Waters Corporation) equipped with a UPLC HSS T3 1.7 µm, 75 µm x 250 mm analytical reverse phase column (Waters Corporation). Prior to peptide separation, 300 ng of tryptic peptides were loaded onto a 2G, V/V 5 µm, 180 µm x 20 mm reverse phase trapping column at 5 µl/min for 3 minutes. IM-DIA-MS analysis of tryptic digests was performed using a Synapt GS-Si mass spectrometer equipped with a T-Wave-IMS device. Mass measurements were made in positive-mode ESI with the instrument operated in resolution mode with a typical resolving power of 20,000 full width at half maximum. Prior to analysis the time-of-flight analyzer was externally calibrated with a NaCsI mixture from *m/z* 50 to 1990. The data were post-acquisition lock mass corrected using the double charged monoisotopic ion of [Glu1]-Fibrinopeptide B. To achieve lock mass correction, a 100 fmol/µl solution of [Glu1]-Fibrinopeptide B was infused at a 90° angle to the analytical sprayer. This reference sprayer was sampled every 60 seconds. Accurate IM-DIA-MS data were collected in the DIA mode of analysis, HDMS^E^ [^24,71^](https://paperpile.com/c/Kjd43B/Vmwi+GEu8) IM spectrometry was performed by applying a constant wave height of 40 V whilst a constant wave velocity of 650 m/s was maintained. Wave heights within the trap and transfer were both set at 4 V whilst the wave velocities were 311 and 175 m/s respectively. MS data were acquired over 50-2000 *m/z for* each mode. Spectral acquisition time for each mode was 0.5 s with a 0.015 interscan delay, corresponding to a cycle of low and elevated energy data being acquired every 1.1 s. During the low energy MS mode data was acquired whilst applying a constant collision energy of 4 eV within the transfer. After IMS, MS/MS data was acquired by ramping the collision energy within the transfer region between 15 and 45 eV. To ensure that ions with a *m/z* less than 350 were derived from peptide fragmentation within the transfer region the radio frequency applied to the quadrupole mass analyser was adjusted to optimise transmission within the region of 350 – 2000 Da. Each biological replicate was analysed at least twice.

### MS Data Processing

All MS data were processed in Progenesis QI for proteomics. Data were imported into Progenesis to generate a 3D representation of the data (*m/z*, RT and peak intensity). Samples were then time aligned with the software allowed to automatically determine the best reference run from the dataset. Following alignment, peak picking was performed on MS level data. A peak picking sensitivity of 4 (out of 5) was set. Peptide features were tentatively aligned with their respective fragment ions based primarily on the similarity of their chromatographic and mobility profiles. Requirements for features to be included in post-processing database searching were as follows: 300 counts for low energy ions, 50 counts for high energy ions and 750 counts for deconvoluted precursor intensities. Subsequent data were searched against 20,049 sequences from the UniProt canonical *Drosophila* database (appended with common contaminants). Trypsin was specified as the enzyme of choice and a maximum of two missed cleavages were permitted. Carbamidomethyl (C) was set as a fixed modification whilst oxidation (M) and N-terminal acetylation were set as variable modifications. Peptide identifications were grouped and relative quantification was performed using non-conflicting peptides only.

## Data

**Supplementary Data 1**

supplementary_data_1.xlsx

Proteomics data

Supplementary Data 2

supplementary_data_2.csv

List of 228 significantly altered proteins and the GMM cluster that they are in

Supplementary Data 3

supplementary_data_3.csv

List of 61 significantly altered proteins in ageing and whether these proteins were also significantly altered by Aβ42

Supplementary Data 4

supplementary_data_4.txt

List of 183 significantly altered proteins contained in a subgraph of the STRING network induced on the 3093 proteins identified by IM-DIA-MS

**Supplementary Data 5**

supplementary_data_5.txt

MCODE modules of significantly altered proteins in Aβ42

**Supplementary Data 6**

supplementary_data_6.xlsx

Gene Ontology enrichment

Supplementary Data 7

supplementary_data_7.txt

MCODE modules of significantly altered proteins in ageing network

## Figures


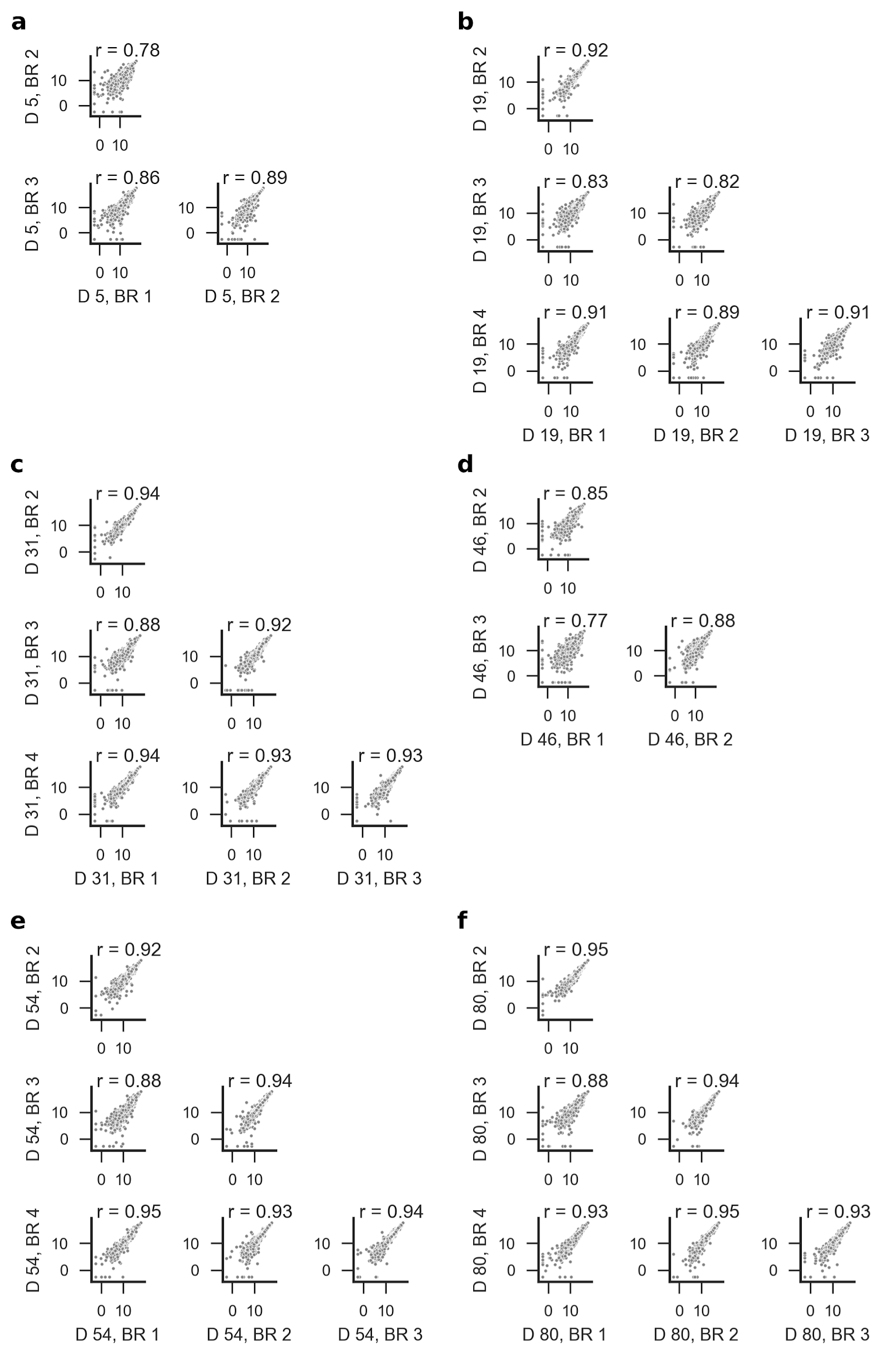


**Figure S1: Assessment of experimental reproducibility.** Scatter plots comparing protein abundances in different biological repeats (BR) of healthy flies at days (D) (**a**) 5, (**b**) 19, (**c**) 31, (**d**) 46, (**e**) 54 and (**f**) 80. Abundances were log2-transformed before plotting. Pearson correlation coefficients (r) are shown for each pair of biological repeat at each time point.


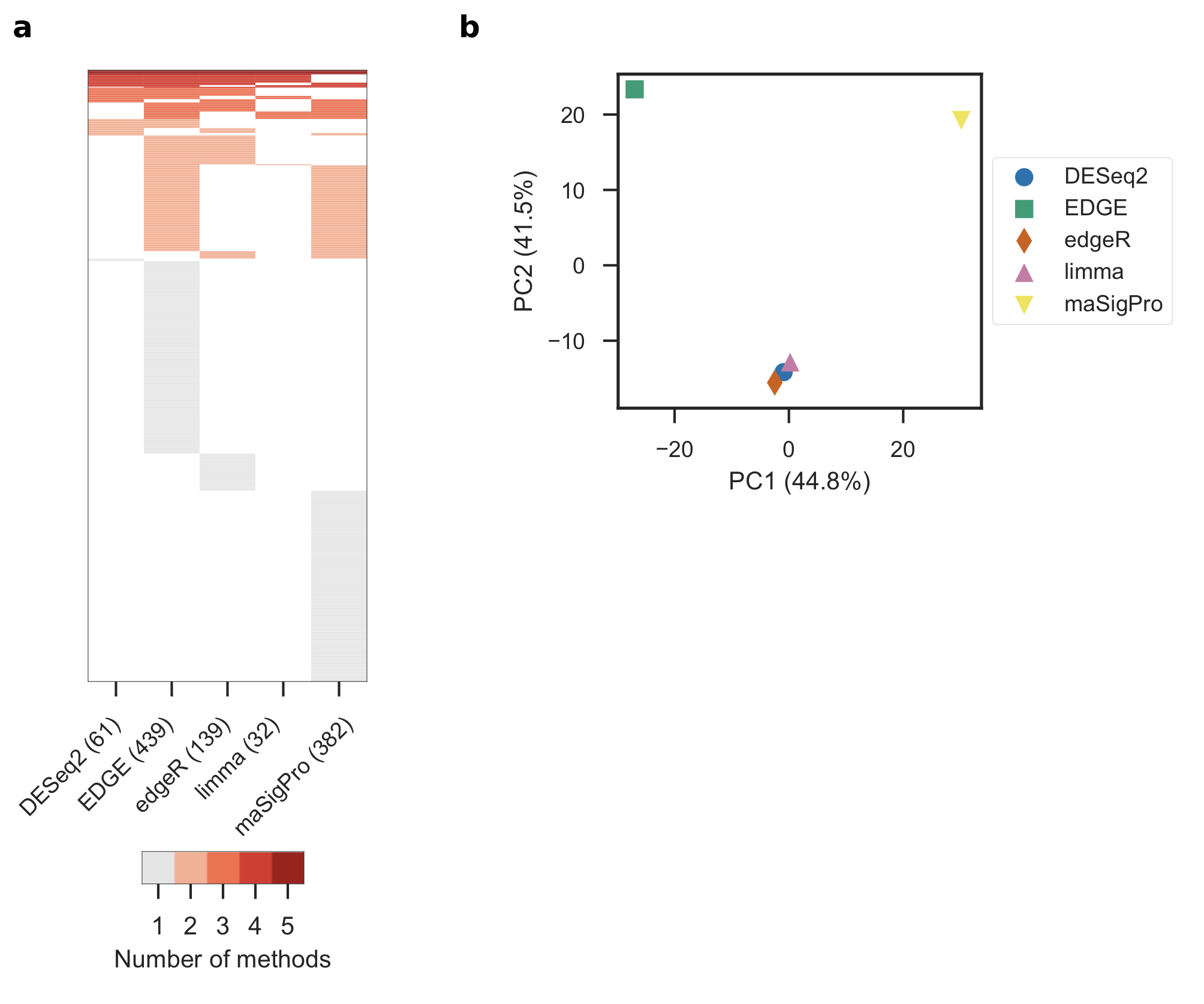


**Figure S2: Analysis of the five statistical methods used to identify significantly altered proteins.** (**a**) Heat map of the proteins detected by each method. (**b**) Principal component analysis of these results. Axes are annotated with the percentage of variance explained by each principal component.


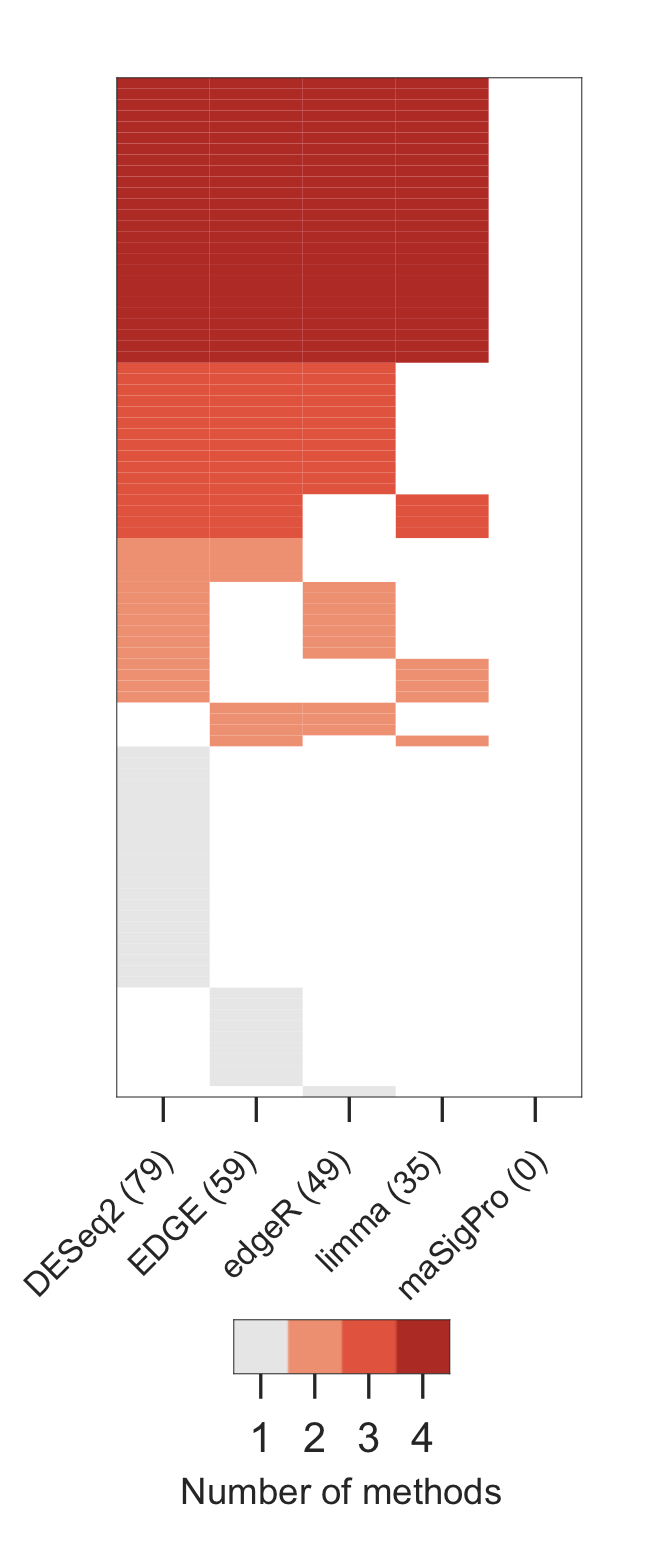


**Figure S3: Identification of significantly altered proteins during normal ageing.** Heat map of the proteins detected by each method.


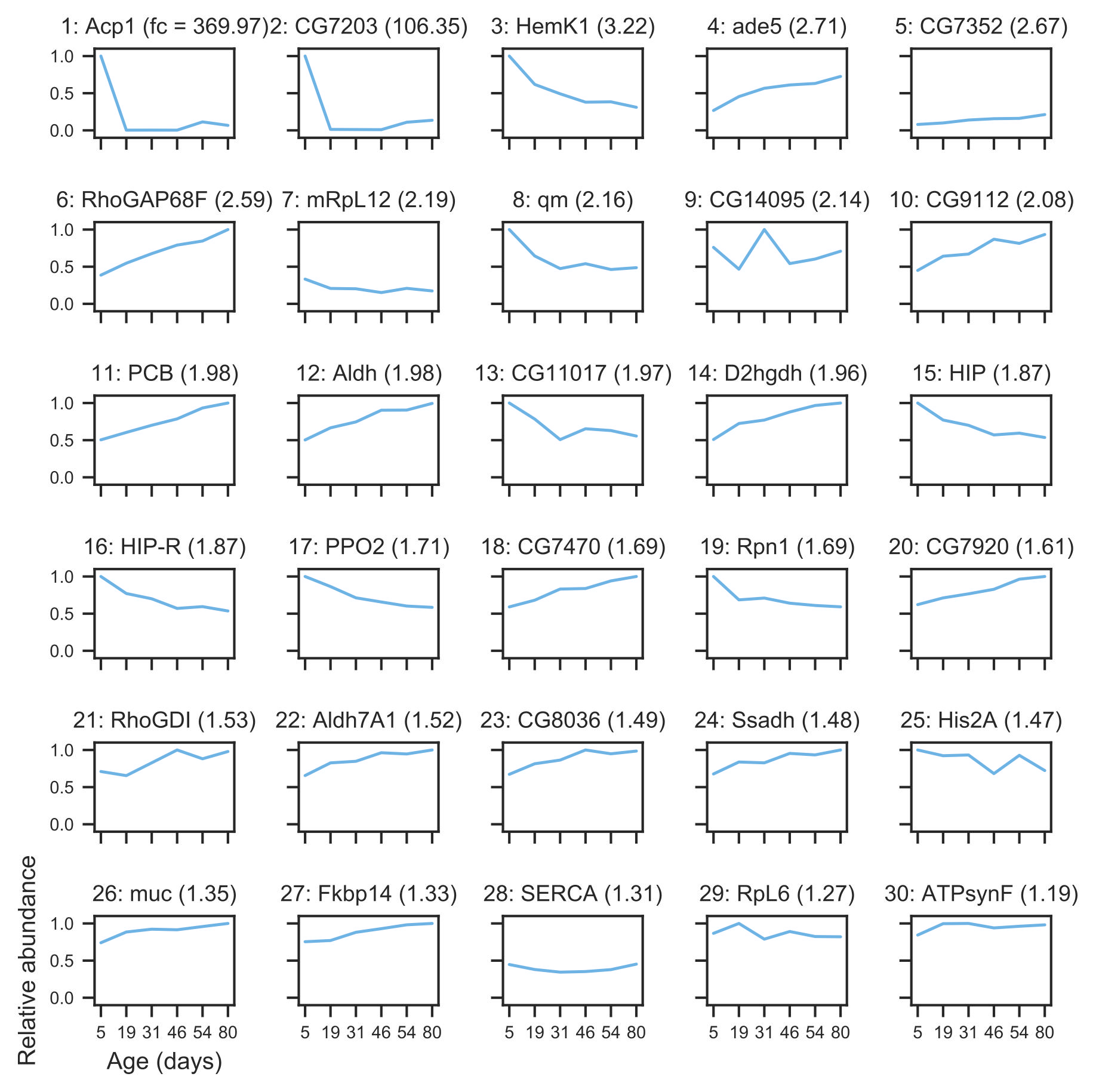


Figure S4: Proteins specifically altered in ageing. Profiles of significantly altered proteins in ageing are shown. Maximum abundances are scaled to 1. Numbers in parentheses denote the maximum observed fold change (fc). Proteins are sorted in descending order of maximum observed fold change.


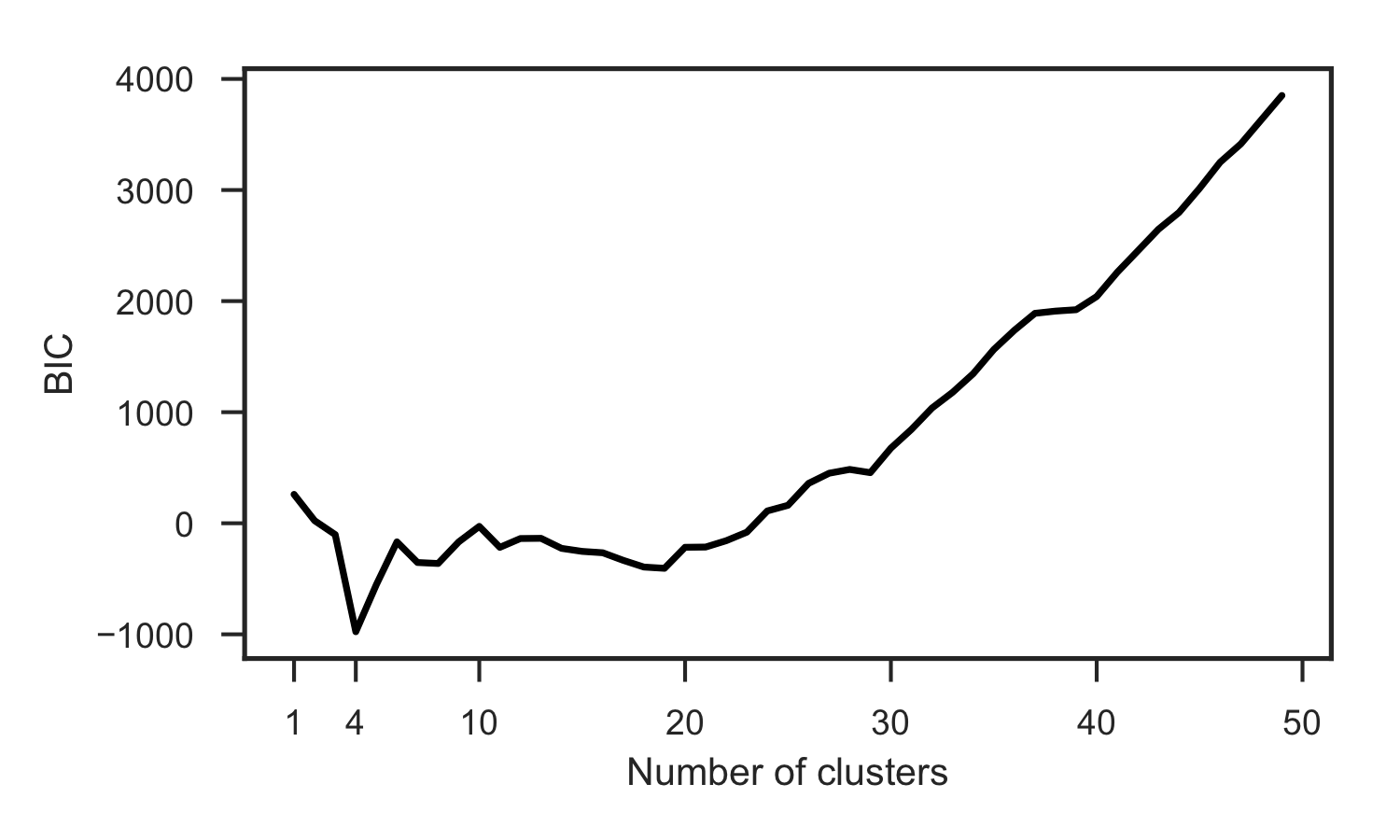


**Figure S5: Model selection for clustering of the significantly altered proteins using a Gaussian mixture model.** The best model was chosen using the Bayesian information criterion (BIC), which penalises complex models.


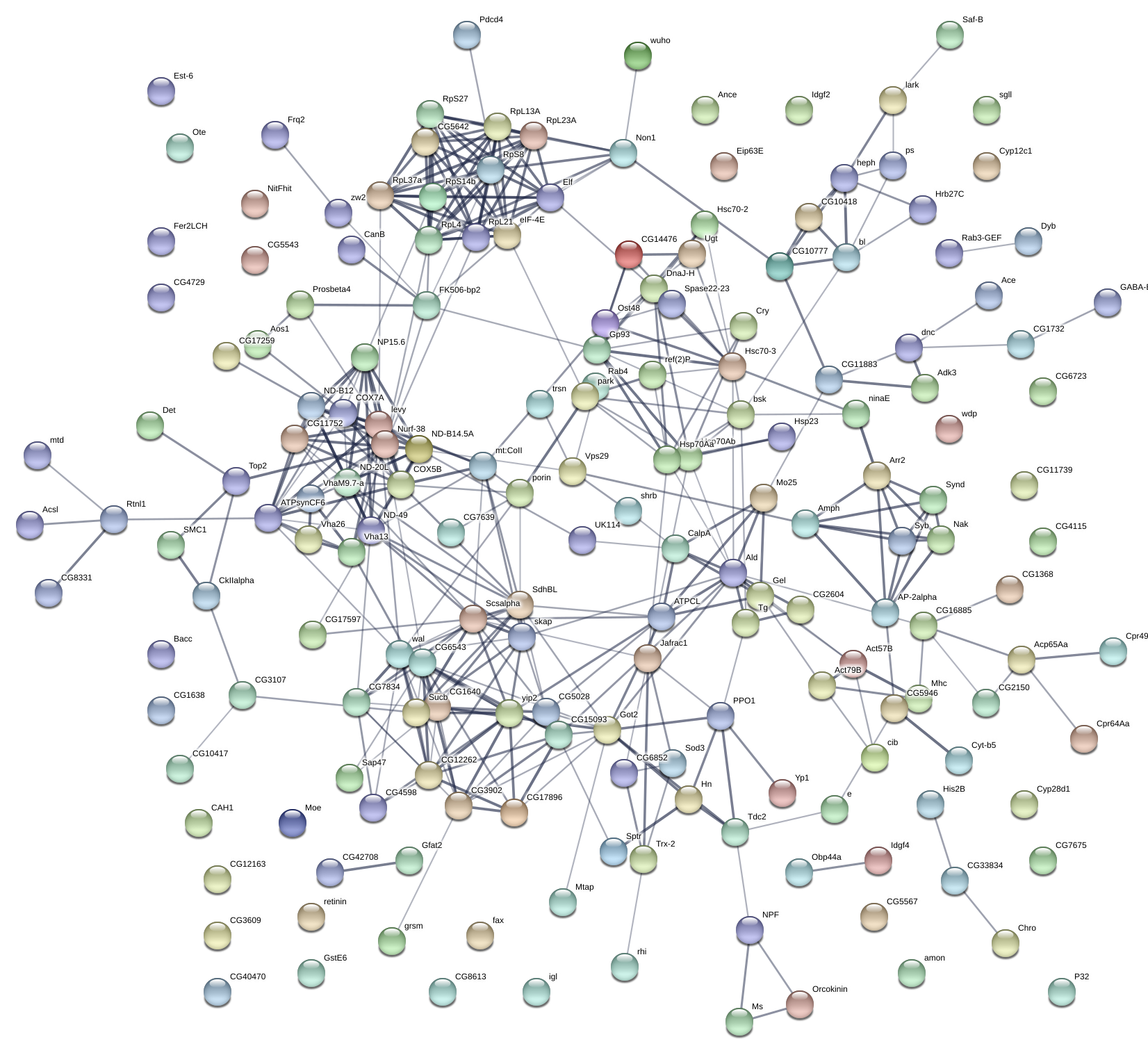


Figure S6: Interactions of the 183 significantly altered proteins present in the STRING network. A subgraph of the STRING network was induced on the 3093 proteins identified by IM-DIA-MS in healthy or Aβ42 flies and the largest connected component was selected (2428 nodes and 44,561 edges). The subgraph contained 183 of the 228 significantly altered proteins (Supplementary Data 3). Edges are weighted by their interaction strength in the STRING database and edges with ‘combined score’ weight < 0.5 have been removed from the network.


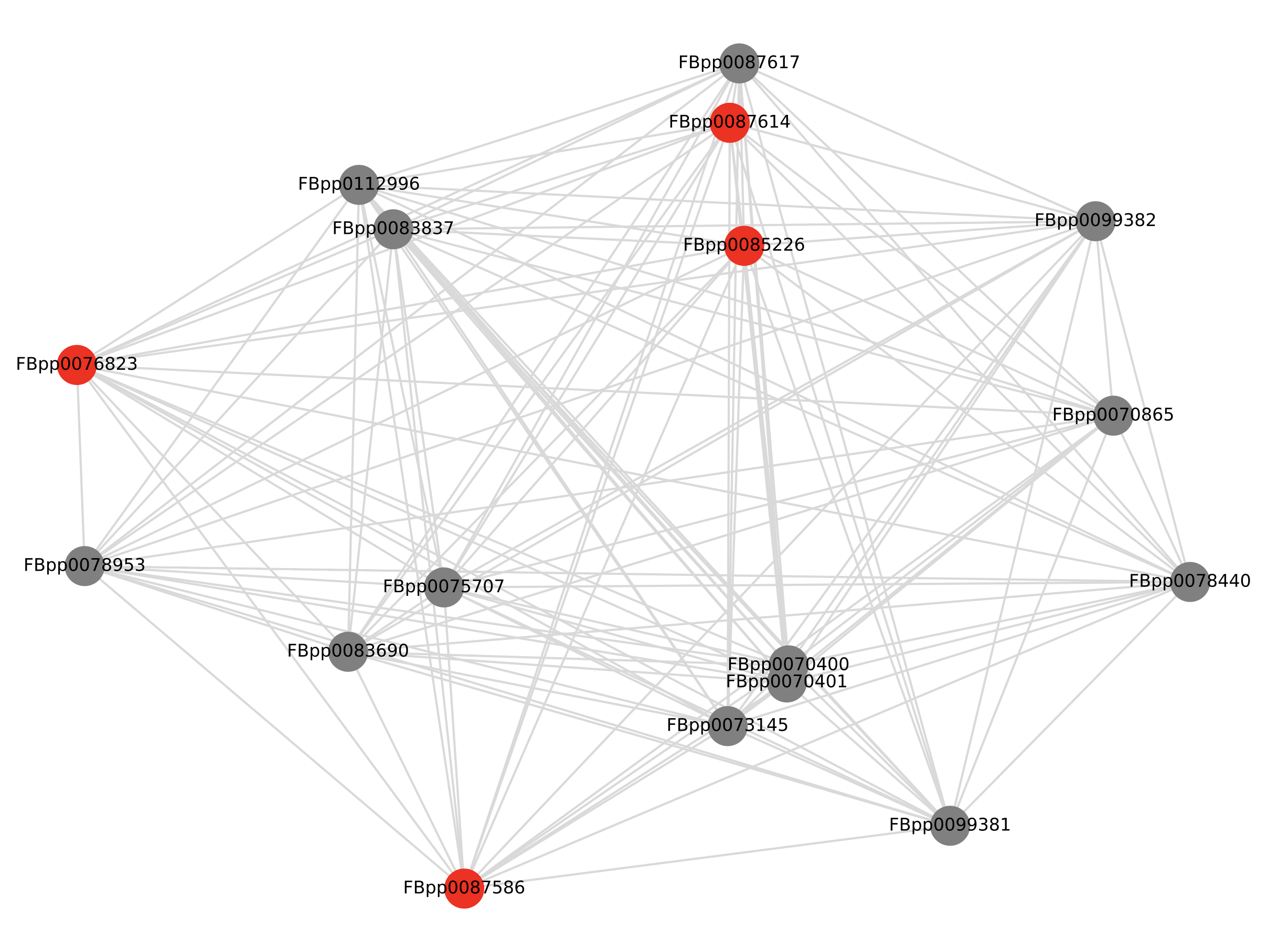


**Figure S7: Network diagram of MCODE module.** Using a subgraph of the STRING network containing the significantly altered proteins and their directly-interacting neighbours, we used MCODE to find modules of densely interconnected nodes. We chose to include neighbouring proteins to compensate for proteins that may not have been detected in the MS experiments due to the stochastic nature of observing peptides and the wide dynamic range of biological samples. The resulting subgraph contained 4842 proteins, including 183 of the 228 significantly altered proteins, as well as 477 proteins that were only identified in healthy or Aβ42 flies and 3125 proteins that were not identified in our IM-DIA-MS experiments. 12 modules were present in the network. This figure details the interactions between proteins in the smallest module (module 8 in Supplementary Data 5). Red nodes denote proteins that are significantly altered in Aβ42 flies.
